# Angiolymphoid Hyperplasia With Eosinophilia Vs Kimura Disease: Continuum of the Same Disease or a Distinct Entity—A Case Report With Literature Review

**DOI:** 10.1155/carm/6601234

**Published:** 2025-11-27

**Authors:** Debananda Sahoo, M. Sai Bodhan, Pavithra Ayyanar, Arpita Dash, Ranjan Kumar Patel, Sujata Devi, Madhusmita Sethy, Anupam Dey

**Affiliations:** ^1^Department of General Medicine, AIIMS, Bhubaneswar 751019, Odisha, India; ^2^Department of Pathology, AIIMS, Bhubaneswar 751019, Odisha, India; ^3^Department of Radiodiagnosis, AIIMS, Bhubaneswar 751019, Odisha, India

**Keywords:** acanthosis, angiolymphoid hyperplasia with eosinophilia, Kimura disease, orthokeratosis, papillomatosis, vasoproliferative disorder

## Abstract

Angiolymphoid hyperplasia with eosinophilia (ALHE) and Kimura disease were previously considered the same entities and are now considered a distinct disorder clinically and histologically. ALHE is a benign vasoproliferative disorder with unclear etiology. The clinical presentation of ALHE includes the involvement of skin and vascular structures sparing lymph nodes. It predominantly involves the head and neck region, extremities, and rarely orbit, oral mucosa, bones, and colon. On the other hand, Kimura disease is a rare benign chronic inflammatory disorder of unknown etiology that predominantly involves subcutaneous lymphoid masses and regional lymph nodes of the head and neck region. Both disorders are classified under hypereosinophilia (HE); however, Kimura disease is more associated with peripheral eosinophilia. It is tough to differentiate both the disorders clinically from each other and also from other HE syndromes including eosinophilic granulomatosis with polyangiitis and systemic HE syndromes. However, tissue diagnosis is the key to differentiation. Here, we describe a female at her 50s without any prior comorbidities, presented to our OPD with atypical multiple symmetrical soft tissue swellings which were of diagnostic dilemmas. She showed features of both ALHE and Kimura disease in investigations. As there is no specific recommendation for treatment, she was started with oral glucocorticoid and weekly methotrexate showing a good response in follow-up visit.

## 1. Introduction

Angiolymphoid hyperplasia with eosinophilia (ALHE) and Kimura disease are organ-specific hypereosinophilic syndromes, usually without systemic manifestations. ALHE occurs in all age groups, peaking in the second to fourth decade with no overall sex predilection. ALHE is a benign vasoproliferative disorder predominantly involving the head and neck region, extremities, and rarely orbit, oral mucosa, bones, and colon, sparing the lymph nodes. In contrast, Kimura disease is a rare benign chronic inflammatory disorder of unknown etiology that predominantly involves subcutaneous lymphoid masses and regional lymph nodes of the head and neck region and occurs in young males, predominantly peaking in the 2^nd^ and 3^rd^ decade of life. This case report is of a female patient in her 50s with an atypical presentation of bilateral symmetric low-flow venous malformations in the auricular region and upper extremities, suggesting a diagnosis of ALHE. On the contrary, regional lymphadenopathy with persistent peripheral blood eosinophilia and elevated IgE suggest Kimura disease. She also had skin, cardiac, and pulmonary involvement along with the cutaneous participation, making it difficult to rule out systemic hypereosinophilic syndromes. Though the above conditions are distinct entities in most cases, there are no definite guidelines to differentiate these entities. Hypereosinophilic syndrome is an umbrella term that includes so many disorders and a continuum of one entity to another is unexplainable. However, tissue diagnosis will help in most conditions [1, 2].

## 2. Case Presentation

A female patient in her 50s with no previously known medical illness came to the General Medicine outpatient department (OPD) with complaints of bilateral symmetric gradually progressive swelling over her posterior arm for the past 4 years. Two years into the illness, she found similar swellings in her bilateral elbow extensor area and left ear lobule area followed by the right ear lobule. She had no constitutional symptoms. Along with these complaints, she also had generalized pruritis, especially over the lesions. She also complained about a Grade II NYHA shortness of breath with occasional cough.

**Figure 1:**
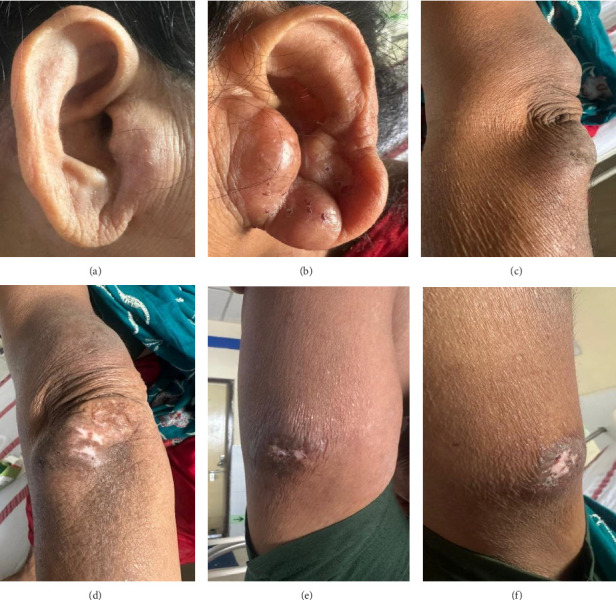
(a–f) Bilateral symmetrical, soft, nonfluctuant, nonreducible, and compressible soft tissue swellings in auricular and upper limb extensor surfaces with hyperpigmentation of overlying skin surfaces (nonpulsatile). (a) Rt ear. (b) Lt ear. (c) Rt elbow. (d) Lt elbow. (e) Rt upper arm. (f) Lt upper arm.

On examination, her vitals were within normal range and auscultation of lungs revealed a decrease in air entry with occasional rhonchi in her bilateral lung fields. The soft tissue swelling (Figures 1(a), 1(b), 1(c), 1(d), 1(e), and 1(f)) is smooth in consistency, nonpulsatile, nonfluctuant, nonreducible, and compressible with overlying hyperpigmented skin with few scratch marks.

### 2.1. Investigations

On investigation, her hemogram showed peripheral blood eosinophilia (AEC-1991{< 500}; differential count; E-24.8%; TLC-8030/cumm), serum IgE-677 IU/mL (2–180 IU/mL), and negative autoimmune profile including ANA IFA and ANCA (both p and c). All other possible causes of hypereosinophilia (HE) are ruled out using stool routine microscopy, serology, and antigen-based tests. Ultrasonography over the lesions revealed slow-flow venous malformations without any pulsatile flow.

**Figure 2:**
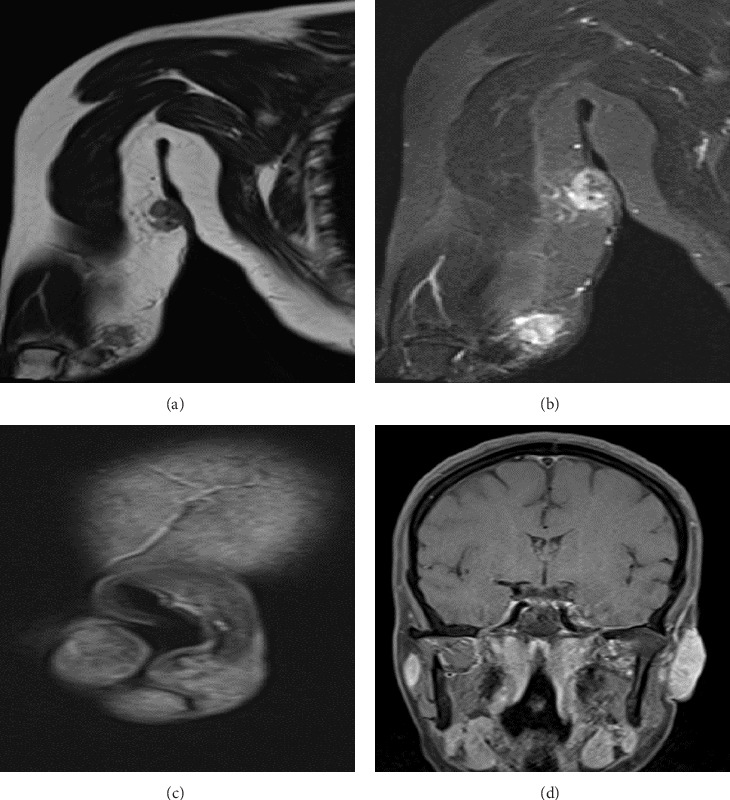
(a–d) MRI of the right arm showed a well-defined T1W hypointense (a) and T2W heterogeneous hyperintense (b) soft tissue lesion with adjacent cystic spaces in the mid 1/3rd of the arm and near the olecranon process. MRI of the face showed that the left ear pinna is swollen and oedematous with diffuse T1/FS hyperintense (c) and T2/TIRM heterogeneous hyperintense (d) soft tissue showing diffusion restriction and intense contrast enhancement.

MRI of the right arm showed a well-defined T1W hypointense (Figure 2(a)) and T2W heterogeneous hyperintense (Figure 2(b)) soft tissue lesion with adjacent cystic spaces seen in the mid 1/3rd of the arm (size ∼1.8 × 2.5 cm), which showed heterogeneous contrast enhancement and patchy diffusion restriction. A similar lesion is seen near the olecranon process of size ∼1.8 × 2.0 cm. A few subolecranon and axillary lymph nodes are seen, the largest being ∼1.4 × 1.8 cm in the right axilla. MRI of the face showed that the left ear pinna is swollen and edematous with diffuse T1/FS hyperintense (Figure 2(c)) and T2/TIRM heterogeneous hyperintense (Figure 2(d)) soft tissue showing diffusion restriction and intense contrast enhancement (size ∼3.8 × 1:7 × 2.9 cm). One small subcentimetric node in the left preauricular region (short axis diameter ∼7 mm) was present. Similar enhancing soft tissue (2.9 × 1.0 cm) and a small lymph node (9 mm) are seen in the right-side pinna also.

Her ECG showed features of a complete left bundle branch block pattern, and echocardiography showed heart failure with midrange ejection fraction (LVEF-42%) and jerky IVS. CAG showed no coronary artery disease; cardiac MRI was done which showed lateral wall hypokinesia with reduced systolic inward excursion of the lateral wall. Holter monitoring showed 3276(4%) VPC burden and 37 bigeminy events. NCS of all 4 limbs showed bilateral Carpel tunnel syndrome of Grade II Modified Hirani (asymptomatic) grade. HRCT thorax showed dependent densities with bronchiectasis and segmental consolidation involving the right middle lobe. PFT showed a restrictive pattern. Allergic broncho pulmonary aspergillosis (ABPA) workup showed no positive result. Bronchoscopy was planned for further evaluation of pulmonary pathology but could not be done due to some logistic issues.

**Figure 3:**
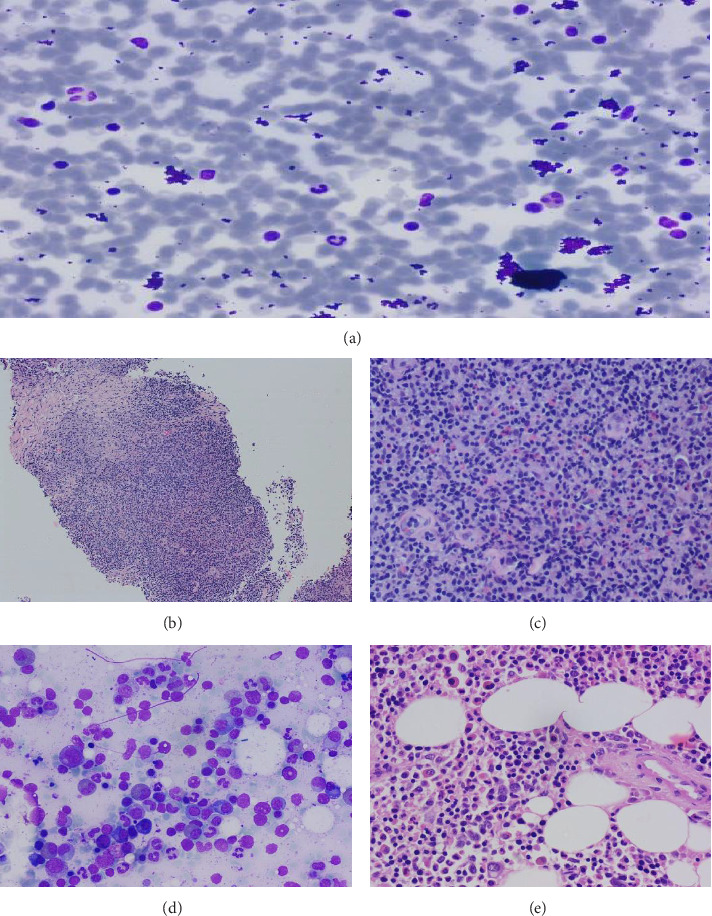
(a–e) Ultrasound-guided fine-needle aspiration cytology (FNAC) done from soft tissue lesions showed (a) smears of reactive lymphoid hyperplasia with scattered eosinophils, plasma cells, and lymphoid cells. Lymph node biopsy ((b)- Core and (c)-20 X microscopy) from left axillary lymph node showed hyperplastic follicles with the germinal center, evidence of folliculolysis by eosinophils, plasma cells, collection of histiocytes, and high endothelial venules are noted. Bone marrow aspiration (d) showed prominence of eosinophils (12%) and eosinophilic precursors. Bone marrow biopsy (e) showed hyper eosinophilia but no evidence of any neoplastic features.

Ultrasound-guided fine-needle aspiration cytology (FNAC) done from soft tissue lesions showed (Figure 3(a)) smears of reactive lymphoid hyperplasia with scattered eosinophils, plasma cells, and lymphoid cells. Lymph node biopsy (Figure 3(b), Core and 3(c), 20 X microscopy) from left axillary lymph node showed hyperplastic follicles with the germinal center, evidence of folliculolysis by eosinophils, plasma cells, collection of histiocytes, and high endothelial venules are noted.

Bone marrow aspiration (Figures 3(d) and 3(e)) showed prominence of eosinophils (12%) and eosinophilic precursors. Bone marrow biopsy showed HE but no evidence of any neoplastic features. Further evaluation with FIP1L1/PDGFRA analysis by FISH was done in peripheral blood, which came out to be negative, ruling out the eosinophilic group of neoplasms.

**Figure 4:**
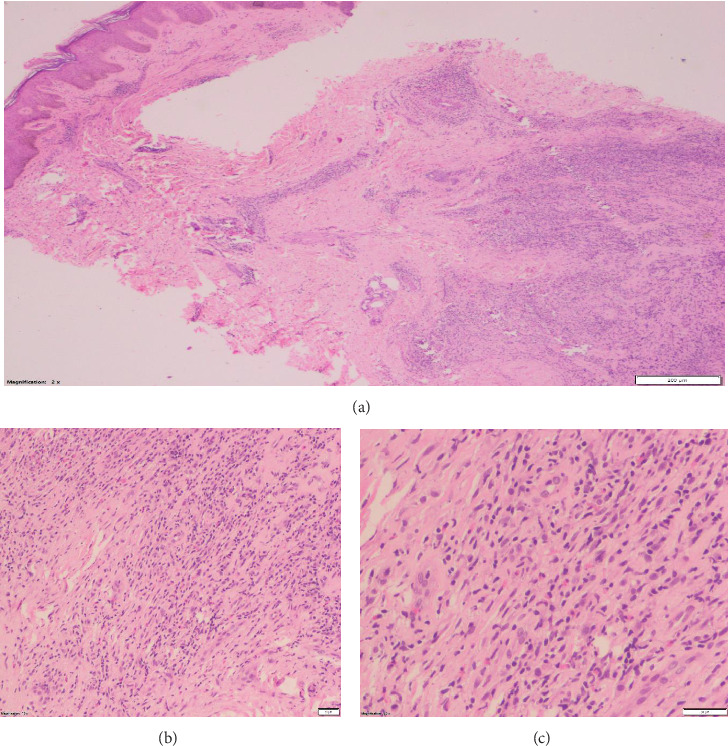
(a–c) Skin biopsy from the pinna and dorsal surface of the forearm showed dense eosinophilic infiltrates, histiocytes, lymphocytes, and plasma cells in the dermis (a), and epidermis showed orthokeratosis, acanthosis, and papillomatosis (b-c) suggestive of angiolymphoid hyperplasia with eosinophilia.

We further proceeded with a skin biopsy (Figures 4(a), 4(b), and 4(c)) from the pinna and dorsal surface of the forearm, which showed dense eosinophilic infiltrates, histiocytes, lymphocytes, and plasma cells in the dermis. Epidermis showed orthokeratosis, acanthosis, and papillomatosis, all suggestive of ALHE.

Table 1 shows features favoring and against the diagnosis of both entities in our patient.

### 2.2. Differential Diagnosis

Eosinophilic granulomatosis with polyangiitis (EGPA) is one of differential for Kimura disease, especially in cases with lung involvement and in ANCA-negative EGPA. Though lung involvement is common in EGPA as in this case, it cannot be used as a stand-alone criterion, and biopsy-proven renal or cutaneous vasculitis would have helped further.

HE syndrome is a systemic disorder whereas Kimura and ALHE are localized forms of HE. However, a boundary cannot be made in few cases where biopsy-proven Kimura or ALHE disease with systemic involvement can present as in this case. A study of bone marrow or peripheral cytogenetics may help in differentiating. Other rare diseases like unicentric Castleman disease, toxoplasma lymphadenitis, Hodgkin's lymphoma, and angioblastic T-cell lymphoma should be considered in appropriate situations.

### 2.3. Treatment

There is no standard guideline-based therapy for ALHA or Kimura disease. Surgical excision is preferred for localized disease. A low-dose radiotherapy postexcision may be used for large lesions to decrease the chance of recurrence. Other treatment approaches include systemic immunosuppressive therapy like oral glucocorticoids, cyclosporine, and mycophenolate mofetil. A few case reports mentioned the use of biological agents like mepolizumab and the interleukin-5 blockade. In ALHE, intralesional glucocorticoids are also used. In our patient, oral glucocorticoids were started along with weekly methotrexate and the option was kept open to start radiotherapy. The patient was also started on ICS, guideline-based management for heart failure with midrange ejection fraction, and antipruritic medication [3, 4].

### 2.4. Outcome and Follow-Up

The patient's general complaints improved significantly after starting therapy. Currently, the patient is being followed up for a response to vascular malformations. Both Kimura and ALHE are benign and localized with not much systemic involvement; however, in our case, there is systemic involvement including cardiac and pulmonary involvement, which needs close monitoring and follow-up. Also, the evolution of disease to another entity is always a possibility as there is systemic involvement.

## 3. Discussion

HE is a common and often ignored finding in both outpatient and inpatient settings. It is defined as the presence of ≥ 1.5 eosinophils × 10^9^/L peripheral blood on two examinations (interval ≥ 2 weeks) with or without tissue eosinophilia. Eosinophils are predominantly tissue-dwelling cells, and their increased number in peripheral blood or tissue can occur in diverse conditions. The classifications of HE (Table 2), various diseases causing HE (Table 3), and various systemic manifestations of HE (Table 4) were described in tabular forms.

Although tissue diagnosis helps in most cases, the main difficulty is to get an adequate tissue sample for processing. Ultrasound, CT, and recent advances like PET-guided biopsy made it simpler in reaching difficult to reach target tissue biopsy sites. Evaluation and management of HE often start with a history and clinical examination, and life-threatening organ manifestations should be dealt with great care. The main principle behind successful treatment is evaluating and management of specific underlying causes.

Western authors have seen these two illnesses as either distinct stages of the same sickness or as part of the same disease [5]. Rosai et al. [6] were the first to identify the histological differences between ALHE and Kimura's illness.

The development of many lymphoid follicles with germinal centers [7–10], many of which are invaded by eosinophils and result in folliculolysis, is the histopathological hallmark of Kimura's illness [7, 10]. Eosinophilic abscesses are typically formed as a result of extensive eosinophilic infiltration [7, 11].

On the other hand, ALHE has more diffuse lymphoid infiltration and sporadic lymphoid follicles. Eosinophilic abscesses are uncommon, while eosinophilic infiltration [10] varies more, ranging from sparse to extensive. When diagnosing recurring Kimura's disease, FNAC may be crucial. Eight cases with a diagnosis of Kimura's disease were examined by Chow et al. [12], who compared the histology description with the FNAC results. They discovered that a backdrop of lymphoid cells, comprising a combination of small and medium-to-large lymphoid cells, was visible in the FNAC smears in every instance of Kimura's disease. The medium-to-large lymphoid cells were formed from the germinal centers of the lymphoid follicles, and these represented cells taken from the lymphoid infiltrates of the lesions. The presence of large numbers of eosinophils, frequently accompanied by eosinophilic granules in their vicinity, was another important characteristic. However, the authors noted that none of the cytological characteristics of Kimura's disease that were stated were unique.

The population of polygonal or spindle-shaped cells with large vesicular nuclei and deeply eosinophilic cytoplasm with well-defined vacuoles, which represent the “epithelioid” or “histiocytoid” endothelial cells observed, is the most noticeable FNAC feature in ALHE, in addition to the background of eosinophils and lymphocytes. Such polygonal or spindle-shaped cells could aid in the FNAC's ability to distinguish between ALHE and Kimura's disease. Based on the cytological findings, further differential diagnoses could be parasite infection, reactive lymphadenopathy with eosinophilia, and, specifically, lymphoma. Therefore, it is recommended to undergo an excision biopsy [12, 13] for confirmation of the initial diagnosis. FNAC may save the patient from undergoing a repeat biopsy examination and is only used to diagnose recurrent lesions in Kimura's illness.

Given the clinical context, our patient's FNAC test from the lesion was compatible with Kimura's disease but the bone marrow biopsy was suggestive of ALHE. It is important to note that our patient had both ALHE and Kimura's disease lesions. Our patient also exhibited peripheral blood eosinophilia and regional lymphadenopathy, which are characteristics more frequently associated with Kimura's illness than ALHE. Although it has been thought that ALHE and Kimura's disease are two distinct disease entities, the evidence from the literature review and the fact that our patient had both ALHE and Kimura's disease lesions suggest that the two conditions may actually be subsets of one another.

Treatment options are very limited in HE without significant underlying causes and organ-specific conditions like Kimura and ALHE as there are no randomized clinical trials. However, most of the cases are treated with steroids, and recurrent cases are often treated with immunosuppressive therapy ± localized radiotherapy [13].

## 4. Conclusion

No boundary limit exists for any disease to present in a particular pattern. HE is a common entity in a medicine outpatient and most of the time, parasitic or atopic conditions are usual causes. A strong suspicion is required in diagnosing rare entities like ALHE and Kimura disease. A thorough investigation is required to rule out all possibilities as HE disorders are not uncommon. Early tissue diagnosis is the key. The fact that the two types of lesions coexist in a single patient may also be proof that ALHE and Kimura's illness are part of the same spectrum.

## Figures and Tables

**Table 1:** Features favoring and against the diagnosis of ALHE and Kimura disease.

**Features suggestive of ALHE**	**Features suggestive of Kimura disease**

Head and neck involvement	Lymph nodal involvement
Vascular involvement	Peripheral blood eosinophilia
Chronic course	Rise in serum IgE
Histopathology of skin biopsy	Histopathology of lymph nodes

**Features against ALHE**	**Features against Kimura disease**

Lymph nodal involvement	Vascular involvement
Peripheral blood eosinophilia	Systemic involvement
Rise in serum IgE	Female gender
Systemic involvement	

**Table 2:** Classification of hypereosinophilia (HE).

Hereditary (familial) HE	Familial clustering, no evidence of reactive, neoplastic diseases, or signs and symptoms of hypereosinophilic syndrome
HE of unknown significance	No evidence of reactive, neoplastic diseases, or signs and symptoms of hypereosinophilic syndrome, no family history
Secondary (reactive) HE	Underlying reactive condition or disease explains HE without other clonal proliferative disorders and hypereosinophilic syndrome features
Clonal (neoplastic) HE	Underlying stem cell, myeloid, or eosinophil neoplasm-inducing HE without hypereosinophilic syndrome features
This classification is published in Allergy by the European Academy of Allergy and Clinical Immunology and John Wiley & Sons Ltd in 2022 [7]	

**Table 3:** Various diseases causing HE.

**Neoplastic diseases**	**Allergic conditions**	**Connective tissue diseases**

Primary (or neoplastic) hypereosinophilic syndromes	Allergic rhinitis	Eosinophilic granulomatous with polyangiitis
Acute eosinophilic leukemia	Chronic rhinosinusitis	IgG4-related diseases
Chronic myeloid leukemia	Asthma	Dermatomyositis
Systemic mastocytosis	Atopic dermatitis	Severe rheumatoid arthritis
B Or T cell lymphoma	**Drug reactions**	Progressive systemic sclerosis
Gastrointestinal adenocarcinoma	Drug reaction with eosinophilia and systemic symptoms (DRESS)	Thromboangiitis obliterans with eosinophilia of the temporal arteries
Squamous cell carcinoma	**Adrenal insufficiency**	Sjogren's disease
**Cholesterol embolization**	**Autoimmune lymphoproliferative syndrome**	Systemic lupus erythematosus
**Radiation exposure**	**Familial eosinophilia**	Behcet syndrome

**Primary immunodeficiency syndromes**		**Secondary immunodeficiencies**

Hyperimmunoglobulin E syndrome		Graft-versus-host disease
Omenn syndrome		Inflammatory bowel disease
Immune dysregulation, polyendocrinopathy, enteropathy, X-linked (IPEX) syndrome		Sarcoidosis
ZAP-70 deficiency		Bullous pemphigoid
		Dermatitis herpetiformis
		Rejection of a transplanted solid organ

*Note:* The bold values specify the headings with broad naming for few disease conditions.

**Table 4:** Various systemic manifestations of HE.

Systemic	Lung	Skin	Gastro intestinal
Eosinophilic granulomatosis with polyangiitis (EGPA)	Eosinophilic asthma	Atopic dermatitis	Eosinophilic esophagitis

Eosinophilic leukemia IgG4-related disease	Eosinophilic granuloma	Urticarial dermatitis	Eosinophilic gastritis

Eosinophilia myalgia syndrome	Eosinophilic bronchitis	Chronic contact dermatitis	Eosinophilic enteritis

Episodic angioedema with eosinophilia (Gleich syndrome)	Eosinophilic pneumonia	Eosinophilic dermatosis of hematologic malignancy	Eosinophil gastrointestinal disorder (EGID)

Acute transplant graft rejections	Simple pulmonary eosinophilia	Ectoparasites	Eosinophilic cholecystitis

Drug reactions	Simple pulmonary eosinophilia	Eosinophilic folliculitis	Eosinophilic colitis

Hematologic malignancy	Occupational lung syndromes	Eosinophilic cellulitis (Wells syndrome)	Eosinophilic pancreatitis

Inborn errors of immunity with eosinophilia	Allergic bronchopulmonary aspergillosis	Eosinophilic ulcer of the oral mucosa	Eosinophilic hepatitis

**Connective tissue**	Tropical pulmonary eosinophilia	T-cell lymphoma	
Eosinophilic myositis		Pemphigoid	

Eosinophilic synovitis	**Naso sinus**	**Others**	

Eosinophilic arthritis	Allergic conjunctivitis (seasonal and perennial)	Eosinophilic ascites	Eosinophilic cystitis

Eosinophilic fasciitis	Giant papillary conjunctivitis	Eosinophilic myocarditis	Eosinophilic prostatitis

**Eye** Eosinophilic Rhinosinusitis	Keratoconjunctivitis (atopic and vernal)	Eosinophilic coronary periarteritis	Eosinophilic endometritis and myometritis (uterus)
Nasal polyposis		Eosinophilic nephritis	Eosinophilic mastitis

*Note:* The bold values specify the headings with broad naming for few disease conditions.

## Patient Perspective

The patient had social and cosmetic concerns regarding the abnormal swellings over the face and upper limb. However, after the diagnosis and initiation of treatment, the patient's symptoms improved and she felt assured.
